# Liver and renal safety of tenofovir disoproxil fumarate in combination with emtricitabine among African women in a pre-exposure prophylaxis trial

**DOI:** 10.1186/2050-6511-15-77

**Published:** 2014-12-24

**Authors:** Justin Mandala, Kavita Nanda, Meng Wang, Irith De Baetselier, Jennifer Deese, Johan Lombaard, Fredrick Owino, Mookho Malahleha, Rachel Manongi, Douglas Taylor, Lut Van Damme

**Affiliations:** FHI 360, 1825 Connecticut Ave, Suite 800, NW, Washington, DC 20009 USA; FHI 360, Durham, NC USA; Institute of Tropical Medicine, Antwerp, Belgium; JOSHA Research, Bloemfontein, South Africa; Impact Research and Development Organization, Kisumu, Kenya; Setshaba Research Centre, Soshanguve, Pretoria, South Africa; Kilimanjaro Christian Medical Center, Kilimanjaro region, Tanzania; The Bill & Melinda Gates Foundation, Seattle, WA USA

**Keywords:** Pre-exposure prophylaxis, HIV, Safety, Women, Africa, Tenofovir disoproxil fumarate, Emtricitabine

## Abstract

**Background:**

Safety of tenofovir disoproxil fumarate/emtricitabine (TDF-FTC) has been studied more extensively among HIV-infected patients than among HIV-uninfected people. Using data from a pre-exposure trial – FEM-PrEP –, we determined the cumulative probabilities of grade 1+ ALT, AST and creatinine and grade 2+ phosphorus toxicities; ALT/AST toxicities by baseline hepatitis B status; and change in mean creatinine, phosphorus, ALT and AST levels controlling for TDF-FTC adherence.

**Methods and findings:**

FEM-PrEP was a randomized, blinded, placebo-controlled trial of daily TDF-FTC among women in Africa. Enrolled women were in general good health, HIV antibody negative, 18 to 35 years old, hepatitis B surface antigen negative, and had normal hepatic and renal function at baseline. AST, ALT, phosphorus and serum creatinine were measured regularly throughout the trial. TDF-FTC concentrations were measured to assess adherence to TDF-FTC. The cumulative probabilities of grade 1+ creatininemia and grade 2+ phosphatemia toxicities were not statistically different between TDF-FTC and placebo arms. The cumulative probabilities of grade 1+ ALT and AST toxicities were higher among participants in the TDF-FTC arm than in the placebo arm (p = 0.03 for both). The proportions of grade 1+ and grade 2+ ALT or AST toxicities were significantly higher in participants who were hepatitis B virus surface antibody (HBsAb) positive than in those who were HBsAb-negative. Women with good adherence had higher mean change from baseline to week 4 in their AST levels (2.90 (0.37, 5.42); p = 0.025) than women with less than good adherence.

**Conclusions:**

We did not observe a significant relationship between randomization to TDF-FTC and creatinine or phosphorus toxicities. Women randomized to TDF-FTC had higher rates of mild to moderate ALT/AST toxicities, especially women with prior hepatitis B virus exposure. We also observed a significant increase in AST from baseline to week 4 among women who had higher adherence to TDF-FTC during that interval.

**Trial register:**

#NCT00625404, February 19, 2008.

## Background

Decades into HIV prevention research, there is strong evidence that the use of antiretrovirals (ARV) as pre-exposure prophylaxis (PrEP) significantly reduces the risk of acquiring HIV infection in men and women [1–5]. While the safety of tenofovir disoproxil fumarate (300 mg)/emtricitabine (200 mg) (TDF-FTC) has been extensively studied among HIV-infected patients, limited data are available among HIV-uninfected people [6–10]. Here we report on the hepatic and renal safety of TDF-FTC in a pre-exposure prophylaxis clinical trial – FEM-PrEP – among HIV negative women in Africa [11].

FEM-PrEP was a randomized, double-blind, placebo-controlled trial of once-daily oral TDF-FTC in Africa among women at high risk of acquiring HIV via sexual intercourse. The study was stopped early due to futility, likely due to low adherence to the daily oral regimen by study participants [11].

The primary safety endpoints included grade 2+ creatinine toxicities and grade 3+ alanine aminotransferase (ALT), aspartate aminotransferase (AST), and phosphorus based on the U.S. NIH, NIAID Division of AIDS (DAIDS) grading table and on local normal ranges [12]. Secondary safety outcomes included grade 1+ ALT, AST and creatinine toxicities and grade 2+ phosphorus. Due to overall low TDF-FTC exposure in the study population, we also conducted exploratory analyses controlling for actual TDF drug levels [11, 13].

We report on the cumulative probabilities of grade 1+ ALT, AST and creatinine toxicities and grade 2+ phosphorus; ALT/AST toxicities by baseline hepatitis B status; and change in means of creatinine, phosphorus, ALT and AST levels controlling for TDF-FTC adherence.

## Methods

### Ethics statement

The trial and informed consent forms were approved by all applicable ethics and regulatory committees: The Protection of Human Subjects Committee of FHI360, the US Food and Drug Administration, the ethics committee (EC) of the University Hospital of Antwerp, the Institutional Review Board of ITM, the Kenyatta National Hospital/University of Nairobi Ethics and Research Committee, the Republic of Kenya Ministry of Health (MoH- Pharmacy & Poisons Board), Kilimanjaro Christian Medical Center Research EC of Tanzania, National Institute for Medical Research of Tanzania, London School of Hygiene and Tropical Medicine EC, Tanzania Food and Drugs Authority, Medunsa Campus Research and EC of South Africa, University of the Free State EC and Medicines Control Council of South Africa. Written informed consent was obtained from all participants prior to conducting any study procedures.

### Context: FEM-PrEP study

The details of the FEM-PrEP study have been described elsewhere [11]. The trial was approved by all applicable ethical and regulatory committees and participants provided written informed consent before any procedures were performed. Women were recruited from Pretoria and Bloemfontein, South Africa; Bondo, Kenya; and Arusha, Tanzania. Eligibility criteria required that women be HIV antibody negative, 18 to 35 years old, in general good health, hepatitis B virus surface antigen (HBsAg) negative and have no evidence of abnormal hepatic or renal function at baseline (i.e., serum creatinine < 1.5 mg/dl, creatinine clearance ≥ 60 ml/min estimated by the Cockcroft-Gault method, ALT and AST <2× local upper limit of normal [ULN] and serum phosphorus levels above the lower limit of the normal range and below DAIDS grade 3). Participants attended study visits at screening, enrollment (2–4 weeks later), and at 4-week intervals thereafter for up to 60 weeks (52 weeks on product followed by eight weeks off product). Plasma and upper layer packed cell (ULPC) aliquots were stored for TDF-FTC concentration testing at monthly visits; hepatic and renal (AST, ALT, creatinine and phosphorus) parameters were evaluated at weeks 4, 12, 24, 36, 52, 56 and when clinically indicated [13].

### Laboratory analysis

AST, ALT, phosphorus and serum creatinine assays were performed according to manufacturer procedures. Samples were processed within two hours after collection. The VITROS DT II instrument (Ortho-Clinical Diagnostics, Inc., Johnson & Johnson, Buckinghamshire, UK) was used in Kenya until May 2010, thereafter the VITROS 250 (Ortho-Clinical Diagnostics, Inc., Johnson & Johnson, Buckinghamshire, UK), the CX5 Beckman Chemistry Analyzer (Beckman Coulter, Inc, Fullerton, CA, USA) was used at both South African sites and the Cobas Integra 400 Plus (Roche Diagnostics GmbH, Mannheim, Germany) was used at the Tanzanian site.

Site-specific laboratory normal ranges (LNR) were used due to unknown population similarities/differences and different instrumentation used across sites. Manufacturer recommended, or nationally/regionally established LNRs were used during the first two to three months of the trial while site-specific LNRs were developed. The local LNR was identified and verified, using Sigma Diagnostic guidelines, by the central laboratory (Institute of Tropical Medicine, Antwerp, Belgium) [14]. Details of the verification process will be described in a separate manuscript. Locally verified ranges were used for final grading of all biochemistry toxicities in Kenya and South Africa. In Tanzania, due to the delay in study initiation and early closure of the trial, local LNR were not verified; manufacturer LNRs were used instead.

The central laboratory provided on-site training in good clinical laboratory practice and technical training for all protocol-required laboratory tests, and conducted quality assurance activities including co-development of site standard operating procedures (SOPs) and analytical plans, assay and equipment validation, external quality assessment (EQA) schemes, and equipment calibration and maintenance. In addition, central lab staff conducted, at a minimum, annual site visits to verify proper conduct of laboratory procedures and review source data.

TDF-FTC concentration analysis on stored samples was conducted at the University of North Carolina at Chapel Hill. Tenofovir (TFV) was measured in plasma and tenofovir diphosphate (TFV-DP) in ULPC. Laboratory procedures for TDF-FTC concentration analysis were published previously [11, 13]. Adherence was qualitatively classified on a 6-point scale based on drug concentration data, with the top two adherence scores being “good” and “excellent” (good = plasma TFV levels >10 ng/ml and ULPC intracellular TFV-DP between 100,000-1,000,000 femtomoles/million cells, excellent = plasma TFV levels >10 ng/ml and ULPC intracellular TFV-DP between >1,000,000 femtomoles/million cells) [13].

### Management of toxicity and study product interruptions

Protocol-defined toxicities were defined and managed according to clinical assessment, grade and relatedness to the study product as assessed by study clinicians [12]. Study product was temporarily or permanently withdrawn as per protocol-described criteria.

Hepatotoxicity grade 2 was defined as 2.6 -5.0 × upper limit of normal (ULN), if determined to be related to the study product, the study product was interrupted and was restarted only if AST/ALT decreased to ≤ grade 1. Hepatotoxicity grade 3 was defined as 5.1 - 10.0 × ULN and study product was interrupted independent of relatedness assessment. Study product was restarted if AST/ALT decreased to ≤ grade 1. Hepatotoxicity grade 4 was defined as > 10.0 × ULN and study product was permanently withdrawn independent of relatedness.

Creatininemia grade 1 was defined as 1.1 -1.3 × ULN and grade 2 as 1.4 -1.8 × ULN; study product was interrupted for both grades regardless of the relatedness to study product use and was restarted if creatininemia decreased to < grade 1 or < 1.3× baseline. Study product was permanently withdrawn for creatininemia grade 2 determined to be related to the study product, and for any creatininemia grade 3 (>1.8 × ULN)

Study product was also permanently withdrawn when there was hypophosphatemia associated with elevated creatininemia, low creatinine clearance or proteinuria. In case of pregnancy, study product was interrupted and resumption allowed after delivery and completion of breastfeeding or with evidence of pregnancy loss/termination). Study product was immediately discontinued in participants who HIV seroconverted.

### Sample selection

The analysis population consisted of all women randomized into the trial, excluding participants who never received study product, returned all product unused, or never returned for a follow-up visit. TDF-FTC concentration data were available for a random sub-cohort of 150 participants assigned TDF-FTC, (50 from each of the three sites where HIV infections took place, i.e., Bloemfontein, Pretoria and Bondo).

### Statistical analyses

Cumulative probabilities of grade 1+ creatinine, ALT and AST and grade 2+ phosphorus were plotted with data pooled across sites by treatment group over time using Kaplan-Meier methods. In addition, the number and of percentage of women experiencing toxicities by grade were summarized by baseline HBsAg status and treatment group; fisher exact tests were used to compare differences between groups.

For the random sub-cohort of 150 women in the TDF-FTC arm, we further assessed the association between adherence to TDF-FTC and change from baseline to week 4 in creatinine, phosphorus, ALT, and AST levels using generalized linear models, with adjustment for baseline level, age, body mass index, oral contraceptive (OC) use at enrollment and study site. A similar analysis was done for change from last visit on-product and first visit after product use ceased (at study exit or earlier product withdrawal). SAS 9.3 (SAS Institute, Cary, NC) was used for all analyses; statistical significance was defined as p ≤ 0.05.

## Results

### Characteristics of enrolled population

The analysis included a total of 2,058 women who were enrolled during June 2009 to April 2011. There were 701 and 707 person-years of follow up in the TDF-FTC and placebo arms respectively. Demographic characteristics, medical history and self-reported sexual behavior at baseline have been reported elsewhere and were generally similar between both groups [11]. At enrolment 20.8% and 21.4% of participants were hepatitis B surface antibody (HBsAb) positive in the TDF-FTC and placebo arms respectively.

### Kidney toxicity

**Figure 1:**
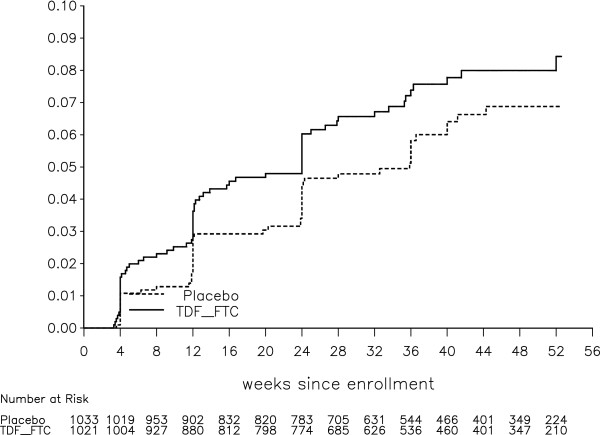
**Cumulative probability of grade 1+ creatinine toxicity, with number of participants at risk at the start of 4-week intervals (log-rank test, stratified on site, p = 0.128).**

**Figure 2:**
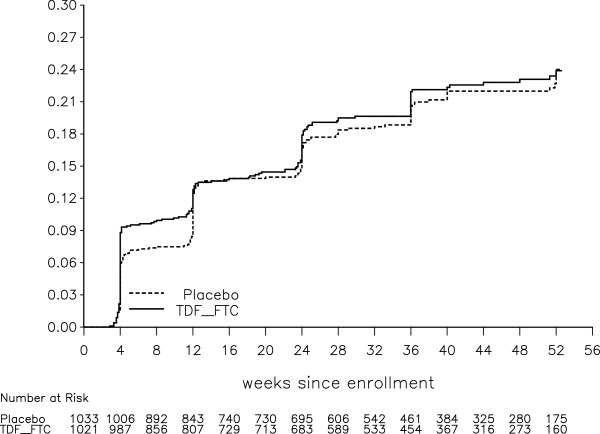
**Cumulative probability of grade 2+ phosphorus toxicity, with number of participants at risk at the start of 4-week intervals (log-rank test, stratified on site, p = 0.621).**

The cumulative probabilities of grade 1+ creatinine or grade 2+ phosphatemia toxicities were higher in the TDF-FTC arm, although neither of these differences were statistically significant (Figures 1 and 2).

Six participants developed grade 2+ creatininemia: four (0.4%) in the TDF-FTC arm and two (0.2%) in the placebo arm. Their baseline creatininemia levels were comparable to the average baseline creatininemia at their respective sites. Among the four participants in the TDF-FTC arm, the grade 2+ creatininemia resolved after 1, 2, 16 and 28 weeks respectively; among the two in the placebo arm, one resolved after 6 weeks and decreased to a grade 1 for 10 weeks in the second participant. The latter participant was referred to a nephrologist at the closing of the trial; she later self-reported that her creatinine returned to normal, though results could not be validated by the study team.

None of the six participants with grade 2+ creatininemia reported taking concomitant medication at the time of the event. TDF-FTC drug concentration data were available for the four participants in the TDF-FTC arm: one was classified as having excellent adherence, two as having good adherence, and one as not adherent in the interval prior to the event.

### Hepatic toxicity

**Figure 3:**
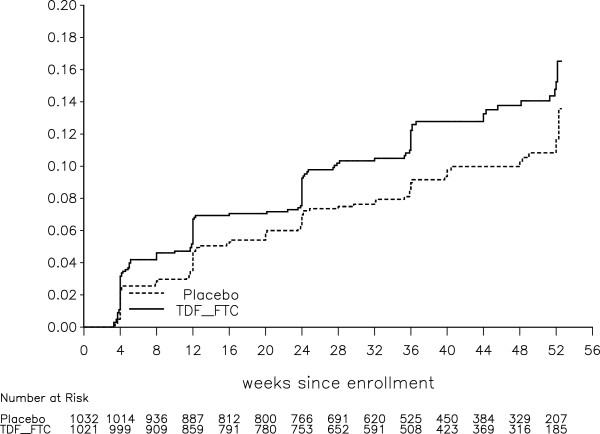
**Cumulative probability of grade 1+ AST toxicity, with number of participants at risk at the start of 4-week intervals (log-rank test, stratified on site, p = 0.025).**

**Figure 4:**
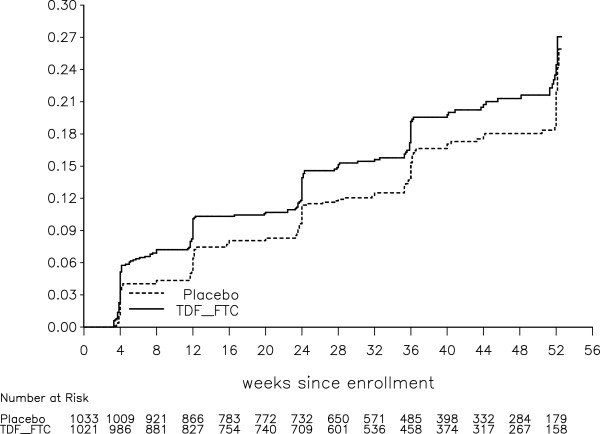
**Cumulative probability of grade 1+ AST toxicity, with number of participants at risk at the start of 4-week intervals (log-rank test, stratified on site, p = 0.025).**

The cumulative probabilities of grade 1+ ALT and AST toxicities were higher among participants in the TDF-FTC arm than in the placebo arm (p = 0.03 for both) (see Figures 3 and 4).

Sixteen participants developed grade 3+ ALT and/or AST toxicities: eight in the TDF-FTC arm and eight in the placebo arm. Baseline AST/ALT levels among these participants were comparable to the average ALT/AST in their respective sites. In three of these 16 participants, the grade 3+ ALT/AST occurred at the time of HIV seroconversion (one in the TDF-FTC arm and two in the placebo arm), and 11 of the 16 (six in the TDF-FTC arm and five in the placebo arm) events occurred in participants who had received the hepatitis B vaccine. Six participants (two in the TDF-FTC arm and four in the placebo arm) with grade 3+ ALT/AST toxicities reported taking a concomitant medication at the time of the event; medications included acetaminophen, ibuprofen, diclofenac and metronidazole. All grade 3+ ALT/AST toxicities were resolved between two and eight weeks without any difference in time to resolution between TDF-FTC and placebo arms. One participant (in the placebo arm) with grade 3+ ALT at week 12 never returned to the study clinic despite intense tracing efforts. Drug level data were available for seven participants in the TDF-FTC arm with grade 3+ ALT/AST: two were classified as having excellent adherence, one as having good adherence and four as non-adherent in the interval before the event.

At one site – Bondo – the proportion of grade 2+ AST toxicities was significantly higher in the TDF-FTC arm (5.0%) than in the placebo arm (1.9%) (p = 0.02). Also, overall, Bondo observed higher proportions of grade 1+ AST toxicities (43.1% in TDF-FTC arm vs. 36.4% in placebo arm) compared to Bloemfontein (5.7% vs. 3.7% respectively), Pretoria (2.9% vs. 2.4% respectively), and Arusha (0% vs. 3.6% respectively). At baseline, Bondo participants had a higher exposure to hepatitis B.

In the TDF-FTC arm, the proportions of grade 1+ and grade 2+ ALT or AST toxicities were significantly higher in participants who were HBsAb positive than in those who were HBsAb negative; for grade 1+, 31.6 vs. 22.4% respectively, p < 0.007 and for grade 2+, 5.6 vs. 2.6% respectively, p < 0.047 (Table 1). In the placebo arm, only the proportion of grade1+ ALT or AST toxicities was significantly more frequent in HBsAb positive participants vs. HBsAb negative participants; 29.5 vs. 17.1%, p < 0.001 (Table 2).

**Table 1:** **Laboratory toxicities during scheduled follow-up in the TDF-FTC arm by previous exposure to HBV**

	HBsAb negative (N=804)					HBsAb positive (N=215)					
	No. of events	No. of women	% of women	Person years	Rate (per 100) person-years	No. of events	No. of women	% of women	Person years	Rate (per 100) person-years	Difference (P-value)#
**ALT or AST**											
Grade 1 or higher	238	180	22.4	449.4	40.05	91	68	31.6	123.4	55.09	9.2 (0.007)
Grade 2 or higher	24	21	2.6	525.0	4.00	12	12	5.6	152.0	7.90	3.0 (0.047)
Grade 3 or higher	6	6	0.7	532.6	1.13	2	2	0.9	156.6	1.28	0.2 (0.678)
Grade 4	1	1	0.1	535.8	0.19	1	1	0.5	157.2	0.64	0.3 (0.378)
**PHO G2+ and creatininemia**											
Grade 1 or higher	11	10	1.2	532.5	1.88	2	2	0.9	156.4	1.28	-0.3 (1.0000)
Grade 2 or higher	0	0	0.0	535.8	0.00	1	1	0.5	156.7	0.64	0.5 (0.2110)
Grade 3 or higher	0	0	0.0	535.8	0.00	1	1	0.5	156.7	0.64	0.5 (0.2110)
Grade 4	0	0	0.0	535.8	0.00	0	0	0.0	157.4	0.00	0 (-)

#Difference in the proportions of participants experiencing toxicities.

**Table 2:** **Laboratory toxicities during scheduled follow-up in the placebo arm by previous exposure to HBV**

	HBsAb negative (N=808)					HBsAb positive (N=224)					
	No. of events	No. of women	% of women	Person years	Rate (per 100) person-years	No. of events	No. of women	% of women	Person years	Rate (per 100) person-years	Difference (P-value)#
**ALT or AST**											
Grade 1 or higher	163	138	17.1	476.1	28.99	79	66	29.5	138.4	47.70	12.4 (0.0001)
Grade 2 or higher	20	19	2.4	527.6	3.60	4	4	1.8	165.0	2.42	-0.6 (0.7996)
Grade 3 or higher	8	8	1.0	531.3	1.51	0	0	0.0	166.4	0.00	-1.0 (0.2126)
Grade 4	2	2	0.2	533.0	0.38	0	0	0.0	166.4	0.00	-0.2 (1.0000)
**PHO G2+ and creatininemia**											
Grade 1 or higher	3	2	0.2	532.6	0.38	0	0	0.0	166.2	0.00	-0.2 (1.0000)
Grade 2 or higher	0	0	0.0	533.3	0.00	0	0	0.0	166.2	0.00	0 (-)
Grade 3 or higher	0	0	0.0	533.3	0.00	0	0	0.0	166.2	0.00	0 (-)
Grade 4	0	0	0.0	533.3	0.00	0	0	0.0	166.2	0.00	0 (-)

#Difference in the proportions of participants experiencing toxicities.

### TDF-FTC concentrations and observed kidney or hepatic toxicities

TDF-FTC concentration data from a sub-cohort of 150 women indicated that very few consistently took the study drug, making it impossible to assess the impact of continuous exposure over longer periods and liver/kidney function. However, the percentage of women with evidence of good adherence in their first four weeks of participation (~40%) was sufficient to assess the impact of short-term drug use. We observed that women with evidence of good adherence had higher mean change from baseline to week 4 in their AST levels (2.90 (0.37, 5.42); p = 0.025) as compared to women with less than good adherence. However, corresponding changes in ALT (1.54 (-1.44, 4.52); p = 0.31), creatinine (0.19 (-2.60, 2.98); p = 0.89) and phosphorus (0.02 (-0.04, 0.08); p = 0.53) were not statistically significant. We did not observe an association between adherence to TDF-FTC and change in ALT, AST, creatinine or phosphorus levels between the final drug-use interval and four weeks after product withdrawal.

## Discussion

In this article we report on change (grade 1+) in renal and hepatic function among African women who were assigned to the active TDF-FTC arm of a randomized, placebo-controlled clinical trial of HIV pre-exposure prophylaxis. While we did not find significant differences in renal functions, we observed a significant increase in the cumulative probabilities of grade 1+ hepatotoxicity, as measured by ALT and AST, among women assigned TDF-FTC.

Our safety findings are comparable to other PrEP studies which also did not observe more frequent renal toxicity in the active arm than in the placebo one [2, 5, 15].

With regard to hepatic toxicity, we observed more frequent elevated ALT or AST in participants with previous exposure to hepatitis B virus; which is consistent with the finding in the Bondo site of significantly more grade 2+ AST toxicities and higher population exposure to hepatitis B virus (39% in Bondo vs. 13%, 10% and 32%, in Bloemfontein, Pretoria, and Arusha, respectively). Additional observations are needed to confirm this preliminary finding, particularly in settings where hepatitis B virus prevalence is high and TDF-FTC will be considered for PrEP. Drug-induced liver injury (DILI) is a possible explanation of our findings. DILI has been described in patients with pre-existing hepatic illness like non-alcoholic fatty liver disease (NAFLD), hepatitis C virus related chronic hepatitis [16, 17]. Our study was not designed to verify association between our observations and any of these specific pre-existing conditions.

None of the laboratory toxicities observed was accompanied by specific renal or hepatic clinical symptoms. The lack of clinical symptoms is likely due to the relatively moderate level of the toxicities observed, frequent testing and subsequent interruption or withdrawal of the study product.

Although these data indicate that the use of TDF-FTC as PrEP is safe with regard to kidney and hepatic toxicity, the results must be considered in light of overall low adherence to study product. Further, our study used the serum creatinine level and the Cockcroft-Gault formula to assess glomerular filtration and phosphatemia to assess proximal tubular function. Some studies have suggested that more sophisticated formulas – e.g. *four-variable Modification of Diet in Renal Disease formula* (MRDR equation) [18, 19] – and multifactor algorithms are more accurate in assessing glomerular filtration and proximal tubular function. In a study comparing tenofovir-containing to tenofovir-sparing HAART in patients living with HIV, Horberg et al., using the MDRD equation, found that the tenofovir-exposed group had a statistically greater percentage of patients with glomerular filtration decline but not creatininemia rising to greater than 2.0 mg/dL [10]. HIV/AIDS patients treated with ARVs can develop kidney and/or liver toxicity for many other reasons than the use of TDF-FTC: HIV infection itself, other ARVs or any other medication to manage their HIV/AIDS condition. In our study, participants were HIV uninfected and healthy at baseline and took limited concomitant medication [20, 21].

Our analysis and conclusions are also limited by short overall TDF-FTC exposure (i.e., follow-up was < = 52 weeks); studies and expert opinions suggest that kidney toxicities may increase over time with prolonged exposure to TDF-FTC [10, 22].

The main limitation of our study is the limited adherence of participants to the study drug; only about 40% exhibited good adherence during any given test interval, and far fewer exhibited consistently good adherence over the course of their participation [11]. Nonetheless, in the TDF-FTC arm we observed higher cumulative probabilities of grade 1+ of ALT/AST and a higher proportion of participants with grade 2+ ALT/AST when previously exposed to HBV.

## Conclusion

In our study population of healthy young African women, we did not observe a significant relationship between randomization to TDF-FTC and creatinine or phosphorus toxicities although we were limited in our ability to adequately assess these relationships due to overall low adherence to the study product. Women randomized to TDF-FTC had higher rates of mild to moderate ALT/AST toxicities, especially women with prior hepatitis B virus exposure. We also observed a significant increase from baseline to week four in AST values among women who were highly adherent to TDF-FTC during that interval.
